# Comparison of immunophenotypes between Rag2 knockout mice derived from two different sources

**DOI:** 10.1186/s42826-023-00153-8

**Published:** 2023-01-11

**Authors:** Yu Jeong Roh, Jeong Eun Gong, Ji Eun Kim, You Jeong Jin, Hee Jin Song, Ayun Seol, Jumin Park, Yong Lim, Dae Youn Hwang

**Affiliations:** 1 Department of Biomaterials Science (BK21 FOUR Program)/Life and Industry Convergence Research Institute/Laboratory Animal Resources Center, College of Natural Resources and Life Science, Pusan National University, Miryang, 50463 Korea; 2 Department of Food Science and Nutrition, College of Human Ecology, Pusan National University, Busan, 46241 Korea; 3 Department of Clinical Laboratory Science, College of Nursing and Healthcare Science, Dong-Eui University, Busan, 47340 Korea

**Keywords:** Rag2, Knockout mice, Immunophenotypes, Spleen, Thymus, T cells, B cells

## Abstract

**Background:**

Recombination activating gene2 (Rag2) knockout (KO) mice are used widely in various research fields, including vaccine development, transplantation studies, and hematopoiesis research, but few studies have compared their phenotypes. This study examined whether there were differences in the immunophenotypes between Rag2 KO mice derived from different sources. In particular, the changes in the organ weight, histological structure, and subpopulation of T and B cells were compared in the spleen and thymus of C57BL/6-Rag2^em1hwl^/Korl (Rag2/Korl KO) and B6.Cg-Rag2^tm1.1Cgn^/J (Rag2/J KO) mice.

**Results:**

The weight of the spleen and thymus similarly decreased in the Rag2/Korl and Rag2/J KO mice compared to their wild type (WT) mice, even though the other organs were kept at the same weight. A slight difference between the Rag2/Korl and Rag2/J KO group were detected in the number of white blood cells (WBC), lymphocytes (LYM), red cell distribution width (RDW), and platelets (PLT). In addition, the white pulp of the spleen and the cortex region of the thymus decreased in both Rag2 KO mice compared to WT mice. On the other hand, significant differences in the number of CD8^+^ T and B cell subpopulations between WT and Rag2 KO mice were observed between Rag2/Korl and Rag2/J KO group, while the CD4^+^ T subpopulation was maintained similarly in both groups.

**Conclusions:**

These results suggest that Rag2/Korl and Rag2/J KO mice exhibit similar immunophenotypes in the spleen and thymus except for the differences in the number of CD8^+^ T and B cell subpopulations.

## Background

**Fig. 1:**
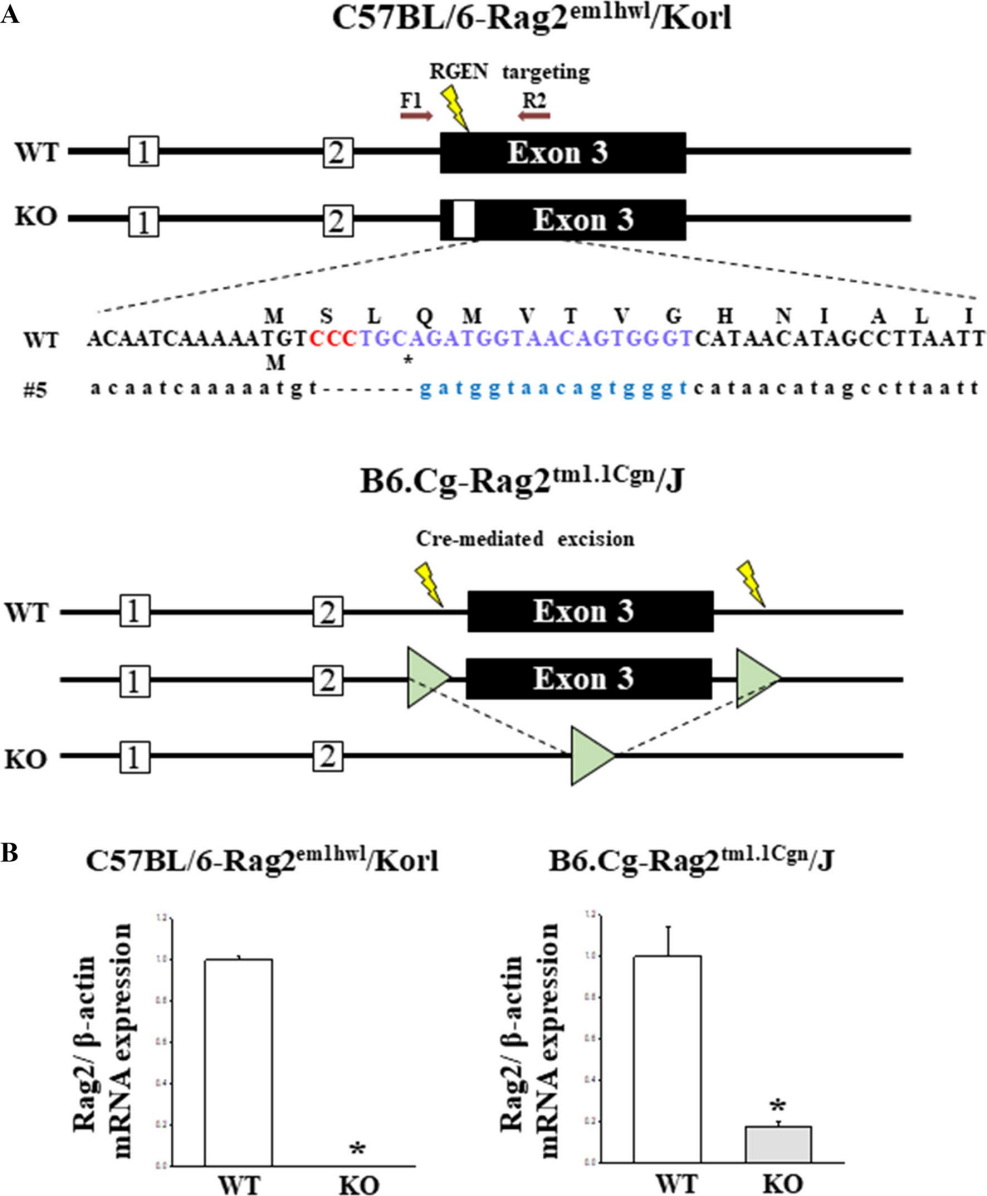
Strategy and conformation for the deletion of the Rag2 gene. **A** Deletion strategy for the Rag2 gene. To generate Rag2/Korl KO mice, a mixture of Cas9 protein and two sgRNA was transferred into a zygote by electroporation, as shown in Materials and Methods. The mice with a 7-nt deletion in exon 3 of the Rag2 gene were used in this study. In Rag2/J KO mice, the Rag2 gene was targeted using the Cre-LoxP system. **B** Expression level of Rag2 mRNA in Rag2/Korl and Rag2/J KO mice. The levels of the Rag2 transcripts were measured in the total mRNA of the bone marrow tissue by RT-qPCR using the specific primers. The mRNA levels of the Rag2 genes were calculated based on the intensity of β-actin as an endogenous control. Four to six mice per group were used to prepare the total RNA; RT-qPCR analyses were assayed in duplicate for each sample. The data are reported as the mean ± SD. *p < 0.05 compared to the WT mice

Rag2 is a critical component of V(D)J recombinase, which mediates the process of V(D)J recombination in variable regions of the immunoglobulin (Ig) and T cell receptor genes during the development of B and T lymphocytes [1, 2]. This protein helps activate the endonuclease functions of Rag1 because it only binds to the DNA strand without endonuclease activity [3, 4]. In addition, Rag2 contributes to the specific recognition of the recombination signal sequences (RSS) to decrease the nonspecific DNA binding by the Rag complex [5]. Based on these functions, a Rag2 deficiency is associated with various diseases, such as severe combined immunodeficiency (SCID), Omenn syndrome (OS), atypical SCID (AS), delayed onset combined immunodeficiency with granulomas and autoimmunity (CID-G/AI), and other delayed-onset atypical presentations [6]. Several animal models with a Rag2 deficiency have been produced using different gene targeting techniques. Early Rag2 deficient mice were generated using a targeting construct that deleted a large portion of the Rag2 coding region. They exhibit phenotypes of severe combined immune deficiency (SCID), including the failure of mature T or B lymphocytes [7]. In addition, B6.Cg-Rag2^tm1.1Cgn^/J mice produced using the Cre-loxP system are used widely to study cancer and toxicology (as a xenograft/transplant host), hematopoiesis, hematology, immunology, and inflammation research because they lack mature lymphocytes [8] (Fig. 1). Recently, a CRISPR/Cas9-mediated gene knockout system was applied to produce two KO mice with different background strains, including C57BL/6Korl and FVB/Korl mice (Fig. 1). Their immunophenotypes, including a lack of mature B and T cells, and an increase in CD45^+^DX-5^+^ natural killer cells, were similar to these of BALB/c-Pkrdc KO mice but not B6-Il2rg KO mice [9]. Furthermore, FVB-Rag2^em1hwl^/Korl mice exhibited hypoplastic changes, severe atrophy in the lymphoid organs, and a loss of mature lymphocytes [10]. On the other hand, there has been no research on the differences and similarities in the immunophenotypes between Rag2/Korl KO mice and commercially provided Rag2/J KO mice with the same C57BL/6 background strain.

This study compared the immunophenotypes of Rag2/Korl KO mice with Rag2/J KO mice derived from different sources to provide sufficient information about mouse models suitable for immunological studies. The alterations in the organ weight, histological structure, and population of T and B cells were analyzed in the spleen and thymus of Rag2/Korl KO and Rag2/J KO mice.

## Results

### Comparison of body and organ weight changes between Rag2/Korl and Rag2/J KO mice

**Fig. 2:**
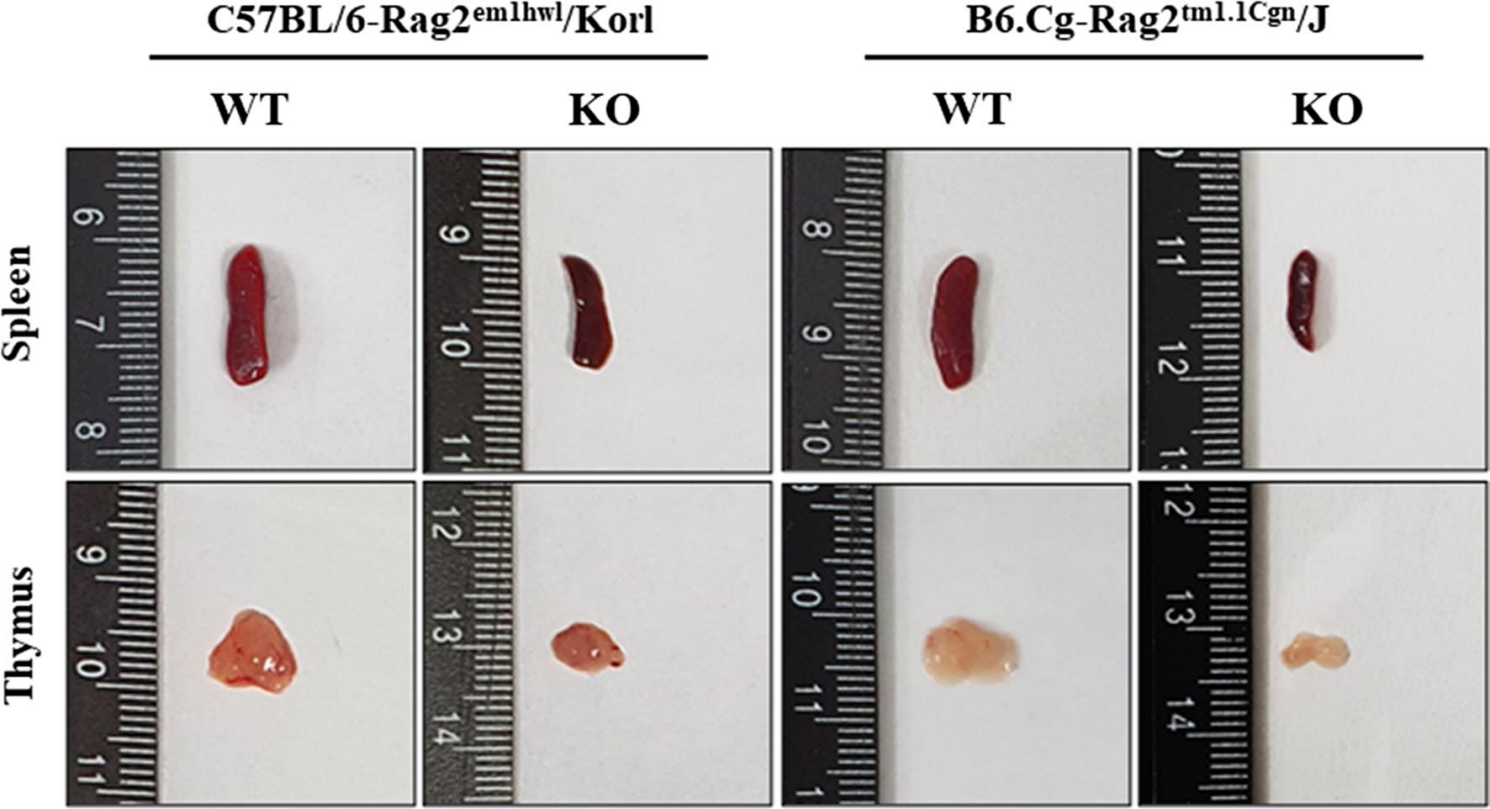
Morphology of the thymus and spleen of Rag2/Korl and Rag2/J KO mice. The thymus and spleen were collected from all mice of the Rag2/Korl and Rag2/J KO groups. Their morphology was observed using a digital camera. Three to five mice per group were used to collect the thymus and spleen, and morphological structure was observed in duplicates for each organ

First, the successful suppression of Rag2 mRNA level was confirmed by RT-qPCR analysis. Their levels were remarkably lower in the bone marrow of both Rag2 KO mice than WT mice (Fig. 1B). This study then examined whether there was a difference in the body weight and organ weight between the Rag2 KO group derived from two different sources. The changes in the body and organ weights were analyzed in Rag2/Korl and Rag2/J KO mice. The difference in body weight between the WT and Rag2 KO mice was not detected in the Rag2/Korl and Rag2/J KO mice group (Table 1). A similar pattern was observed in the weight of most organs, including the liver, kidneys, lungs, and heart, except for two immune organs. The weights of the spleen and thymus were significantly lower in the Rag2 KO mice than in the age-matched WT mice. Their decrease rate was greater in the Rag2/J KO group (− 68.44% and − 68.80%, respectively) than in the Rag2/Korl KO group (− 43.72% and − 62.06%, respectively) (Table 1 and Fig. 2). Hence, there are some differences in the weight loss of the spleen and thymus between the Rag2/J KO and Rag2/Korl mice.

**Table 1:** Body and organ weight of Rag2/Korl and Rag2/J KO mice

Category	C57BL/6-Rag2^em1hwl^/Korl			B6.Cg-Rag2^tm1.1Cgn^/J		
	WT	KO	Change ratio (%)	WT	KO	Change ratio (%)
Body weight (g)	26.90 ± 1.57	24.73 ± 1.26	−7.88 ± 6.19	26.47 ± 1.40	26.90 ± 1.01	+ 3.98 ± 10.13
Liver weight/Body weight (%)	4.90 ± 0.07	4.92 ± 0.18	+ 5.13 ± 9.08	4.94 ± 0.36	4.48 ± 0.31	−2.09 ± 12.68
Kidney weight/Body weight (%)	1.27 ± 0.07	1.35 ± 0.06	2.78 ± 16.72	1.17 ± 0.04	1.13 ± 0.02	−0.70 ± 3.46
Spleen weight/Body weight (%)	0.34 ± 0.05	0.17 ± 0.01*	−43.72 ± 13.51	0.28 ± 0.03	0.10 ± 0.02*	−68.44 ± 16.28^#^
Lung weight/Body weight (%)	0.64 ± 0.04	0.78 ± 0.05*	+ 19.15 ± 15.47	0.63 ± 0.01	0.70 ± 0.04*	10.54 ± 7.49
Thymus weight/Body weight (%)	0.17 ± 0.05	0.07 ± 0.02*	−62.06 ± 17.13	0.15 ± 0.02	0.09 ± 0.05*	−68.80 ± 7.54
Heart weight/Body weight (%)	0.54 ± 0.06	0.54 ± 0.04	+ 9.43 ± 12.65	0.48 ± 0.03	0.53 ± 0.05	6.93 ± 6.22

### Comparison of the blood analysis parameters of the Rag2/Korl and Rag2/J KO mice

This study investigated whether there was a difference in the blood analysis parameters between the Rag2 KO groups derived from two different sources. The changes in the levels of 12 parameters in the whole blood of Rag2/Korl and Rag2/J KO mice were analyzed. Among them, six parameters, including WBC, LYM, NEU, HGB, and PLT, differed remarkably between the WT and Rag2 KO mice. Only four parameters, including WBC, LYM, RDW, and PLT, exhibited a significant difference between Rag2/Korl and Rag2/J KO groups. In particular, the decrease rates in the number of WBC and LYM were greater in the Rag2/J KO group (− 84.58% and − 96.26%, respectively) than in the Rag2/Korl KO group (− 58.62% and − 93.04%, respectively). The RDW and PLT levels showed an opposite pattern; the increase and decrease in the level of each parameter were reversed (− 3.03% and 4.09% for the Rag2/J KO group and 27.72% and − 17.56% for the Rag2/Korl KO group) (Table 2). Hence, there are some differences in the WBC, LYM, RDW, and PLT levels between the Rag2/Korl and Rag2/J KO groups but the other blood analysis factors remain constant.

**Table 2:** Alteration on the blood analysis parameters of Rag2/Korl and Rag2/J KO mice

Category	C57BL/6-Rag2^em1hwl^/Korl			B6.Cg-Rag2^tm1.1Cgn^/J		
	WT	KO	Change ratio (%)	WT	KO	Change ratio (%)
WBC (× 10^3^ cells/μL)	4.01 ± 0.29	1.70 ± 0.26*	−58.62 ± 6.58	4.92 ± 1.56	0.73 ± 0.15*	−84.58 ± 3.65^#^
LYM (× 10^3^ cells/μL)	3.39 ± 0.17	0.26 ± 0.06*	−93.04 ± 1.84	4.30 ± 1.19	0.15 ± 0.02*	−96.26 ± 1.68^#^
NEU (× 10^3^ cells/μL)	0.37 ± 0.11	1.38 ± 0.20*	+ 280.28 ± 146.16	0.20 ± 0.06	0.56 ± 0.07*	+ 262.46 ± 43.67
RBC (× 10^6^ cells/μL)	9.29 ± 0.82	8.74 ± 0.25	−5.94 ± 11.05	9.25 ± 0.41	8.78 ± 0.43	−5.02 ± 5.32
HGB (g/dL)	12.33 ± 0.33	11.1 ± 0.34*	−9.10 ± 0.73	12.48 ± 0.33	11.53 ± 0.25*	−5.58 ± 4.33
HCT (%)	39.93 ± 0.98	36.42 ± 0.76*	−8.41 ± 0.42	40.01 ± 1.52	37.65 ± 1.33	−4.74 ± 5.22
MCV (fL)	40.50 ± 0.58	40.80 ± 0.84	+ 0.83 ± 2.83	42.0	42.2 ± 0.45	+ 0.48 ± 1.06
MCH (pg)	12.88 ± 0.37	12.55 ± 0.39	−2.28 ± 4.65	13.08 ± 0.36	13.13 ± 0.35	−0.38 ± 6.85
MCHC (g/dL)	30.94 ± 1.63	30.73 ± 1.89	−0.43 ± 8.41	31.03 ± 0.95	31.03 ± 0.88	−0.50 ± 7.20
RDW (%)	17.82 ± 0.42	17.25 ± 0.46	−3.03 ± 1.57	17.28 ± 0.27	17.98 ± 0.54	+ 4.09 ± 4.30^#^
PLT (× 10^3^ cells/μL)	490.67 ± 38.08	654.25 ± 4.27*	+ 27.72 ± 2.00	489.67 ± 17.90	432.3 ± 4.51*	−17.56 ± 10.26^#^
MPV (fL)	6.13 ± 0.06	5.95 ± 0.10	−1.64 ± 2.32	5.86 ± 0.06	5.77 ± 0.15	−1.36 ± 2.81

### Comparison of histopathological changes between Rag2/Korl and Rag2/J KO mice

**Fig. 3:**
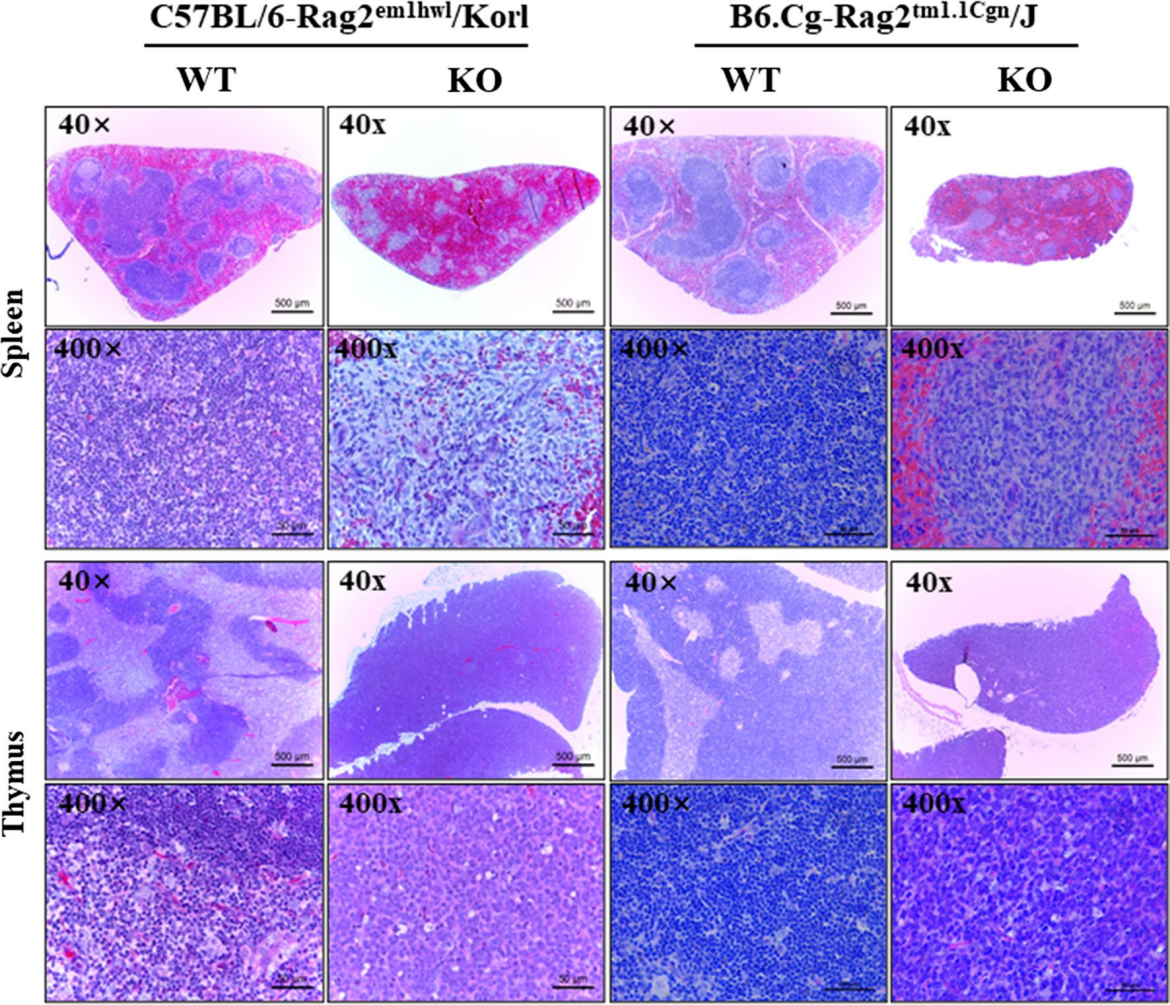
Histopathological structure of the spleen and thymus. After preparing the hematoxylin and eosin (H&E)-stained sections, alterations on their histopathological structures were observed in the spleen and thymus of the Rag2/Korl and Rag2/J KO mice at 100× and 400× magnification using an optical microscope. Three to five mice per group were used in the histological analysis, and histopathological structure was observed in duplicates in two different slides

The changes in the histopathological parameters were analyzed in the H&E-stained spleen and thymus of Rag2/Korl and Rag2/J KO mice to determine the differences in the histological structure of immune organs between the Rag2 KO group derived from two different sources. In the spleen, an area of white pulp was significantly lower in the Rag2 KO mice than in the WT mice, while red pulp was enlarged in the same group. In addition, the number of lymphocytes was lower in the Rag2 KO mice than in the WT mice. These alterations were similar in the Rag2/Korl and Rag2/J KO groups (Fig. 3). In the thymus, most of the medulla region disappeared in the Rag2 KO mice compared to the WT mice, but there was no significant difference between Rag2/Korl and Rag2/J KO groups (Fig. 3). Therefore, histopathological analyses suggested that there is no difference in the structure of spleen and thymus between the Rag2/Korl and Rag2/J KO group.

### Comparison of the CD4^+^ T cells in the spleen and thymus of the Rag2/Korl and Rag2/J KO mice

**Fig. 4:**
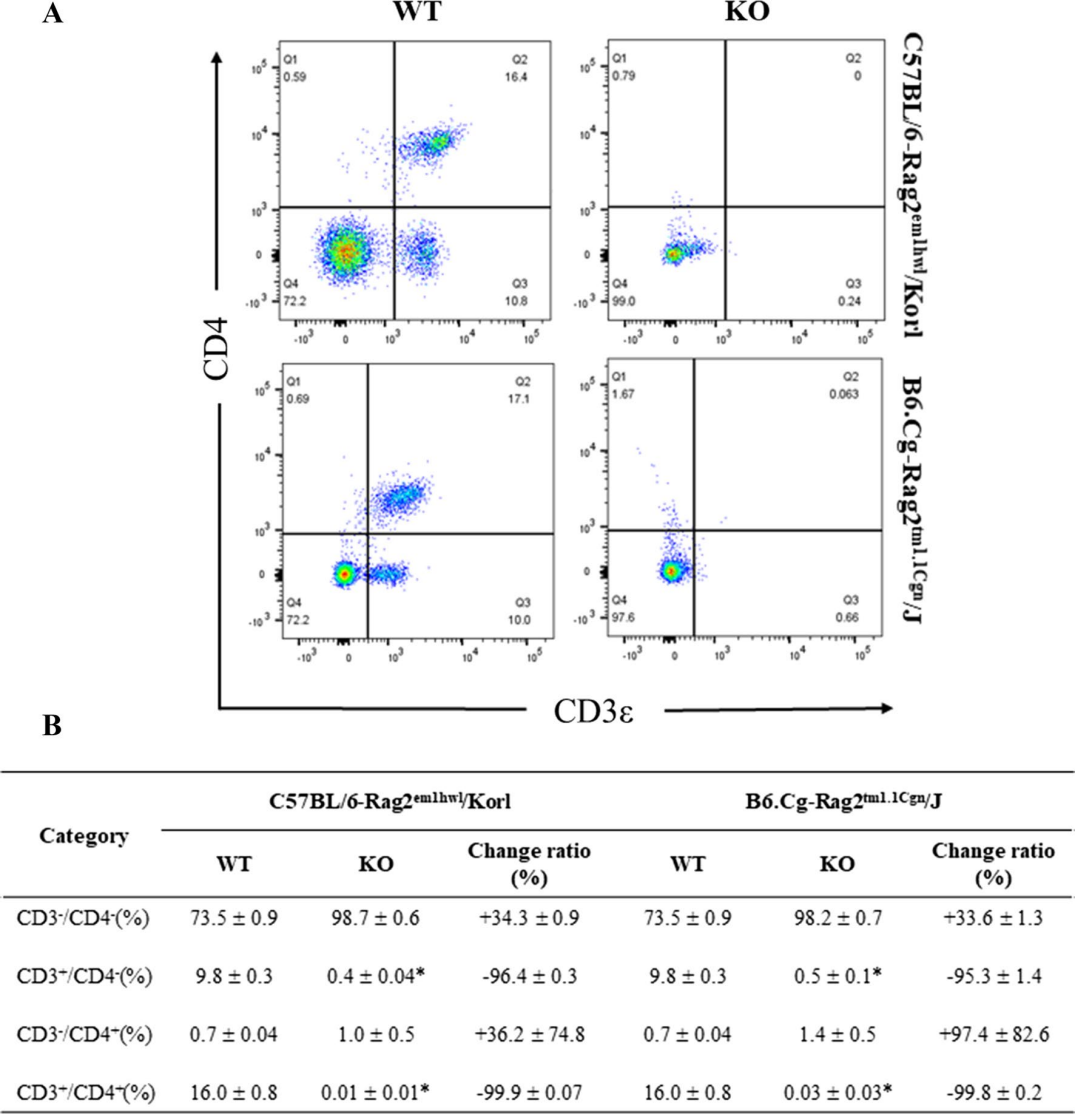
FACS analysis of T_h_ cells in the spleen of the Rag2/Korl and Rag2/J KO mice. The total cells were isolated from the spleen and stained with anti-CD3 and anti-CD4 antibodies, as described in the materials and methods. The distributions of the double negative (DN), single positive (SP), and double positive (DP) cell populations were analyzed by flow cytometry. Three to five mice per group were used to prepare and stain the total spleen cells, and the distribution of the cell population was analyzed in duplicate. The data are reported as the mean ± SD. *p < 0.05 compared to the WT mice

**Fig. 5:**
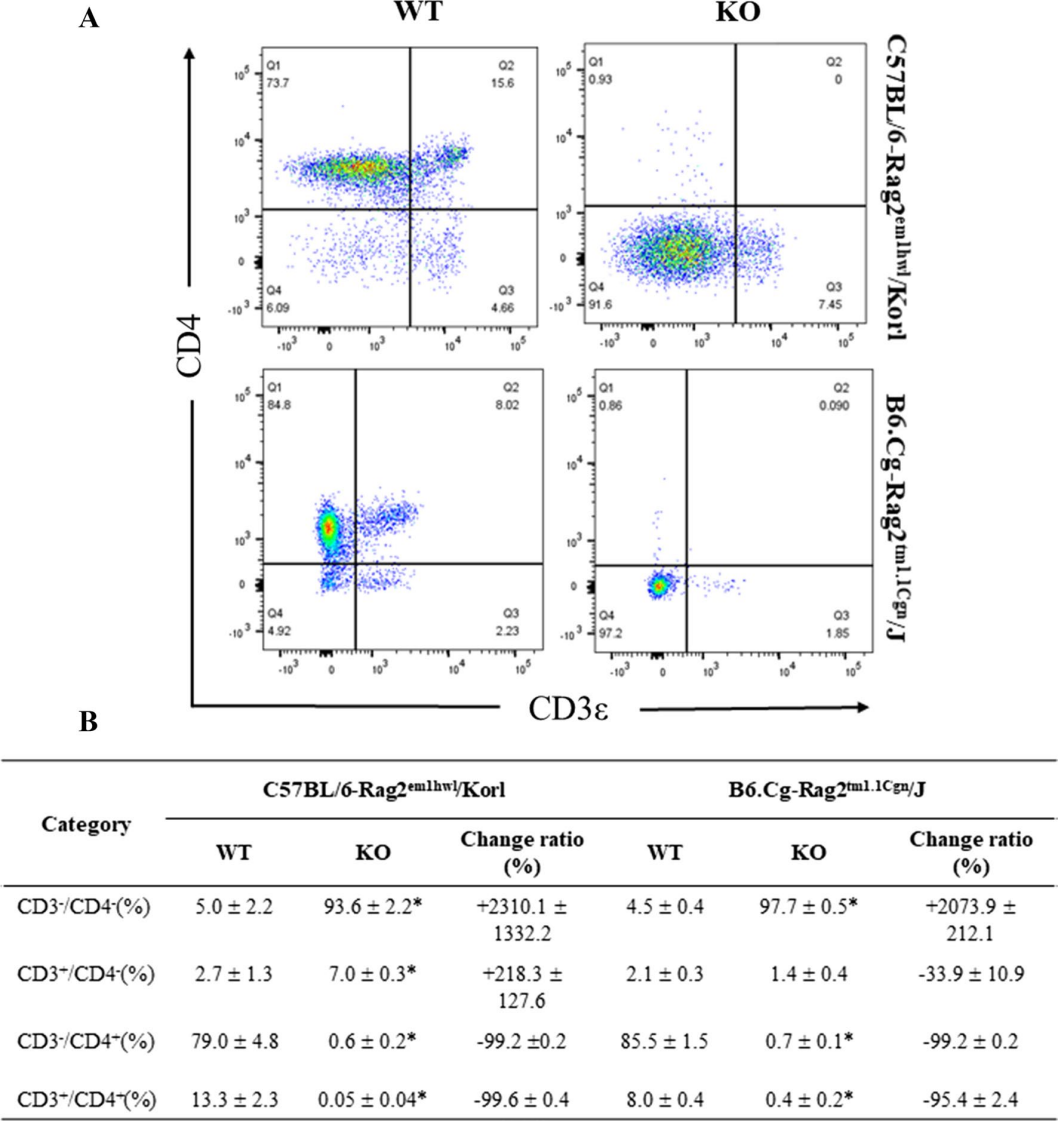
FACS analysis of T_h_ cells in the thymus of Rag2/Korl and Rag2/J KO mice. The total cells were isolated from the thymus and stained with anti-CD3 and anti-CD4 antibodies, as described in the materials and methods. The distributions of the double negative (DN), single positive (SP), and double positive (DP) cell populations were analyzed by flow cytometry. Three to five mice per group were used in the preparation and staining of total thymus cells, and the cell population distribution was analyzed in duplicate. The data are reported as the mean ± SD. *p < 0.05 compared to the WT mice

This study investigated whether there was a difference in the subpopulation of CD4^+^ T cells of immune organs between the Rag2 KO group derived from two different sources. The numbers of four subpopulations of CD4^+^ T cells (CD3^−^/CD4^−^, CD3^+^/CD4^−^, CD3^−^/CD4^+^, and CD3^+^/CD4^+^) were counted in the cells isolated from the spleen and thymus of the Rag2/Korl and Rag2/J KO mice after staining the anti-CD3 and anti-CD4 antibodies. In the spleen, some significant changes in the CD3^+^/CD4^−^ and CD3^+^/CD4^+^ T cells were detected in the Rag2 KO mice than in the WT mice. The number of these two subpopulations was significantly lower in the Rag2 KO mice, but there was no difference between the Rag2/Korl and Rag2/J KO mice (Fig. 4). On the other hand, the thymus showed very different patterns in the distribution of the CD4^+^ T cell subpopulation compared to the spleen. Among the four subpopulations, the number of CD3^−^/CD4^−^ T cells was significantly higher in the Rag2 KO mice (93.6% and 97.7%, respectively) than in the WT mice (5.0% and 4.5%, respectively), while the number of CD3^−^/CD4^+^ (79.0% to 0.6% and 85.5% to 0.7%, respectively) and CD3^+^/CD4^+^ T cells (13.3% to 0.05% and 8.0% to 0.4%, respectively) decreased in the same groups. These differences between WT and KO mice were constant in the Rag2/Korl and Rag2/J KO group (Fig. 5). Hence, there is no distinct difference in the CD4^+^ T cell subpopulation of the spleen and thymus between the Rag2/Korl and Rag2/J KO groups.

### Comparison of CD8^+^ T cells in spleen and thymus between Rag2/Korl and Rag2/J KO mice

**Fig. 6:**
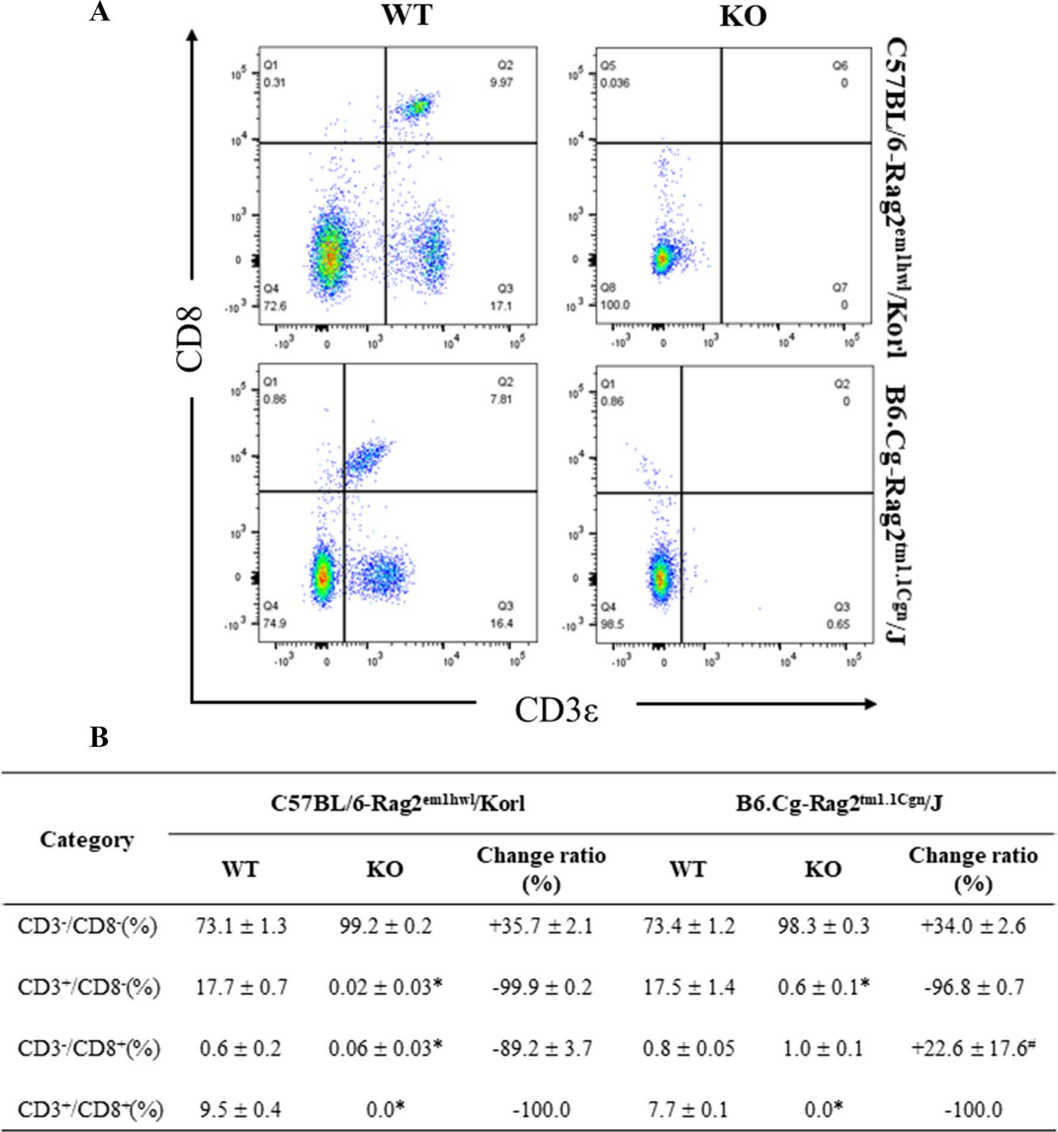
FACS analysis of T_c_ cells in the spleen of Rag2/Korl and Rag2/J KO mice. The total cells were isolated from the spleen and stained with anti-CD3 and anti-CD8 antibodies, as described in the materials and methods. The distributions of the double negative (DN), single positive (SP), and double positive (DP) cell populations were analyzed by flow cytometry. Three to five mice per group were used in the preparation and staining of the total spleen cells, and the distribution of the cell population was analyzed in duplicate. The data are reported as the mean ± SD. *p < 0.05 compared to the WT mice. ^#^*p* < 0.05 compared to the change ratio of Rag2/Korl KO mice

**Fig. 7:**
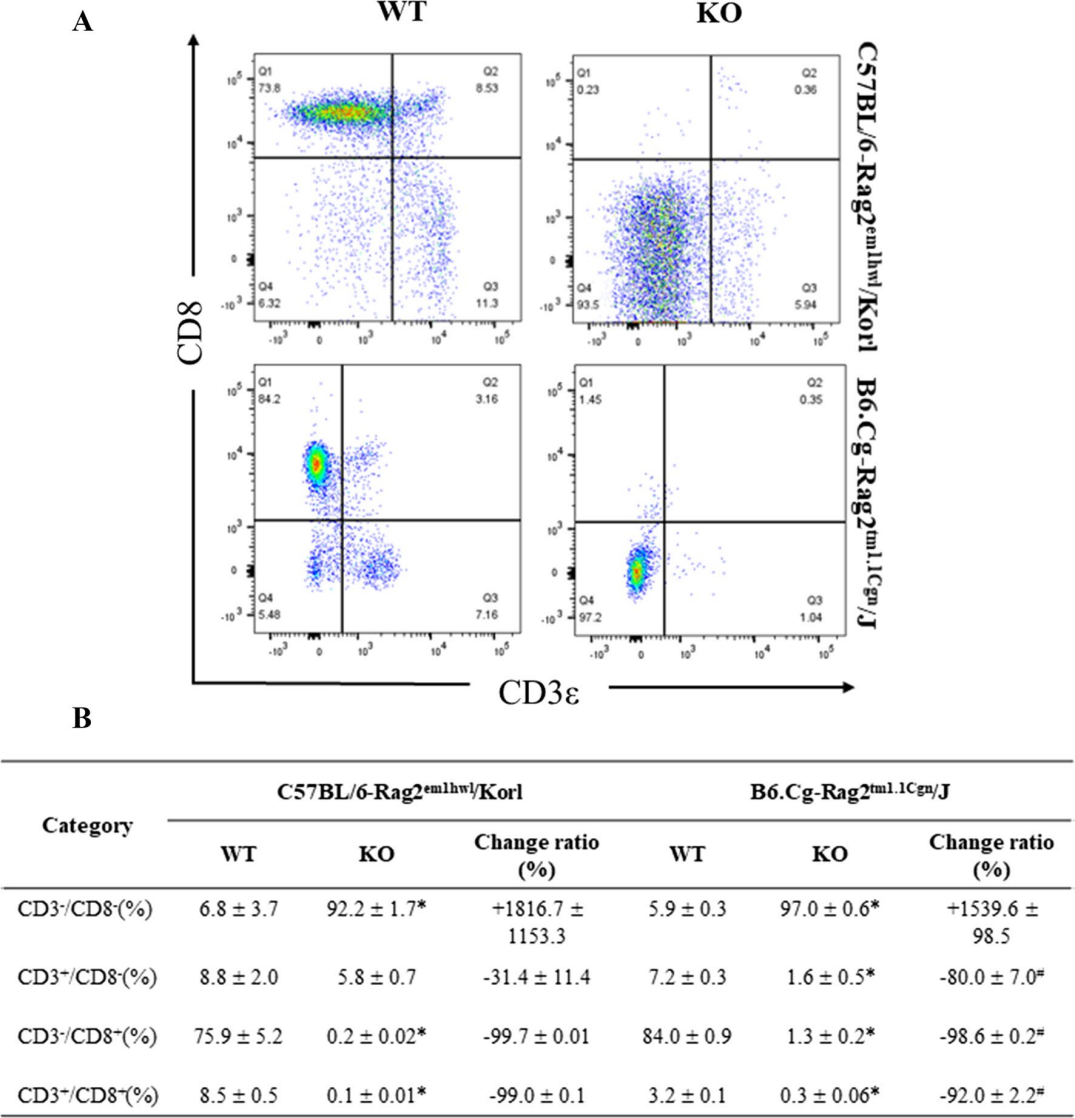
FACS analysis of T_c_ cells in the thymus of Rag2/Korl and Rag2/J KO mice. The total cells were isolated from the thymus and stained with anti-CD3 and anti-CD8 antibodies, as described in the materials and methods. The distributions of the double negative (DN), single positive (SP), and double positive (DP) cell populations were analyzed by flow cytometry. Three to five mice per group were used in the preparation and staining of the total thymus cells, and the distribution of the cell population was analyzed in duplicates. The data are reported as the mean ± SD. *p < 0.05 compared to the WT mice. ^#^*p* < 0.05 compared to the change ratio of Rag2/Korl KO mice

Experiments were conducted to determine if there was a difference in the subpopulation of CD8^+^ T cells of immune organs between the Rag2 KO group derived from two different sources. The numbers of four subpopulations of CD8^+^ T cells (CD3^−^/CD8^−^, CD3^+^/CD8^−^, CD3^−^/CD8^+^, and CD3^+^/CD8^+^) in the cells isolated from the spleen and thymus of Rag2/Korl and Rag2/J KO mice were counted after staining with anti-CD3 and anti-CD8 antibodies. In the spleen, a significant difference in CD3^−^/CD8^−^, CD3^+^/CD8^−^, and CD3^+^/CD8^+^ T cells was detected between WT and Rag2 KO mice. Among these, the difference between Rag2/Korl and Rag2/J KO mice was observed in only the CD3^+^/CD8^−^ T cells. The rate of decrease in these numbers was slightly higher in the Rag2/Korl KO group (–99.9%) than in the Rag2/J KO group (− 96.8%) (Fig. 6). In the thymus, the number of the three cell subpopulations (CD3^−^/CD8^−^, CD3^−^/CD8^+^ and CD3^+^/CD8^+^) were significantly different in the WT and Rag2 KO mice. The CD3^−^/CD8^−^ T cell subpopulation was significantly higher in the Rag2 KO mice than in the WT mice, while the number of CD3^−^/CD8^+^ and CD3^+^/CD8^+^ T cells was lower in the same group. Significant differences in the CD3^−^/CD8^+^ (− 99.7% to − 98.6, respectively) and CD3^+^/CD8^+^ T cell subpopulation (− 99.0% to − 92.0%, respectively) were detected in the Rag2/Korl and Rag2/J KO groups. Nevertheless, this difference between Rag2/Korl and Rag2/J KO group was extremely low despite being significant (Fig. 7). Hence, there is a very small difference in the distribution of CD8^+^ T cell subpopulation from the spleen and thymus the Rag2/Korl and Rag2/J KO group.

### Comparison of B cells in spleen between Rag2/Korl and Rag2/J KO mice

**Fig. 8:**
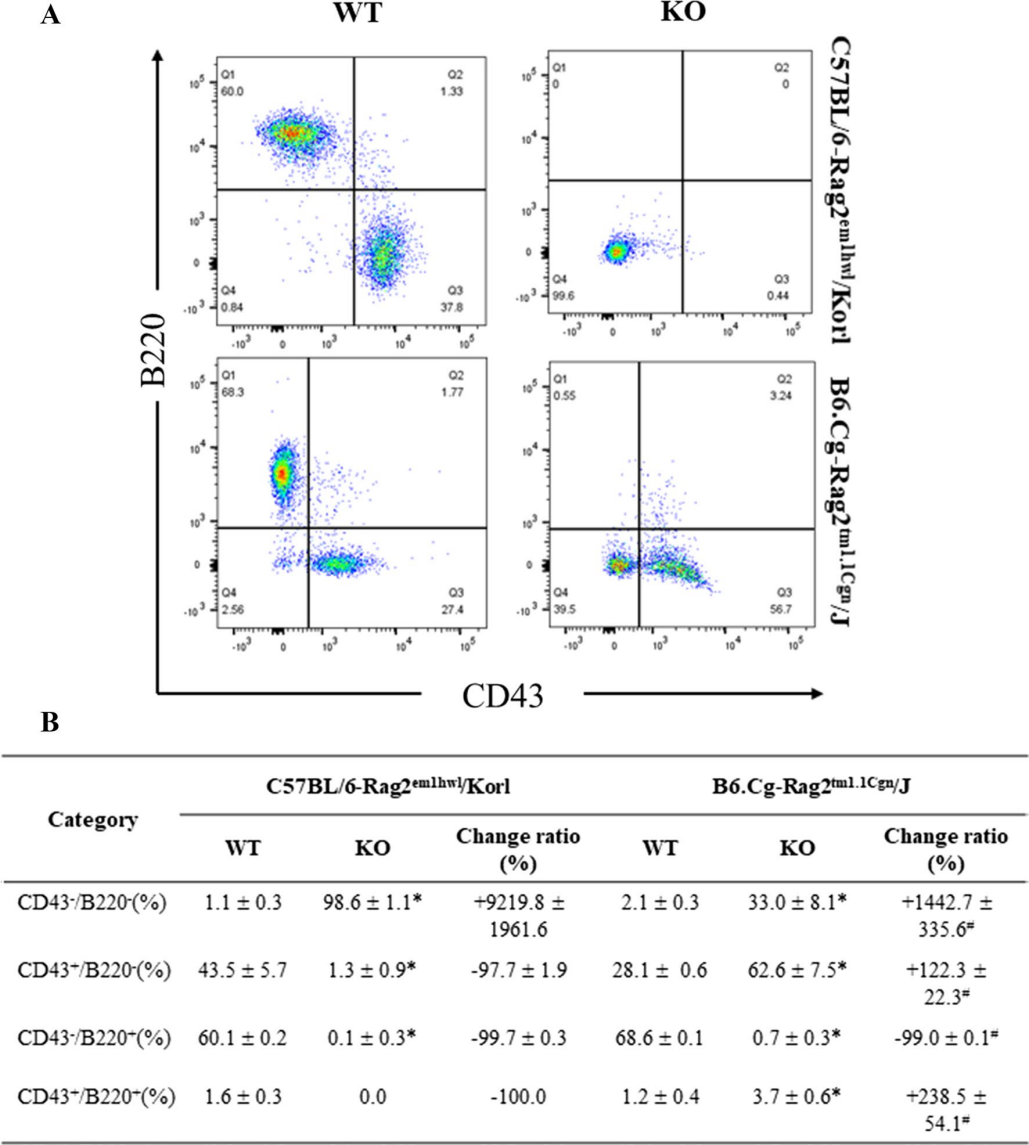
FACS analysis of B cells in the spleen of Rag2/Korl and Rag2/J KO mice. The total cells were isolated from the spleen and stained with anti-CD43 and anti-B220 antibodies, as described in the materials and methods. The distributions of the double negative (DN), single positive (SP), and double positive (DP) cell populations were analyzed by flow cytometry. Three to five mice per group were used to prepare and stain the total spleen cells, and the distribution of the cell population was analyzed in duplicate. The data are reported as the mean ± SD. *p < 0.05 compared to the WT mice. ^#^*p* < 0.05 compared to the change ratio of Rag2/Korl KO mice

Finally, this study examined whether there was a difference in the distribution of B cells in the spleen between WT and Rag2 KO groups derived from two different sources. The CD43^−^/B220^−^, CD43^+^/B220^−^, CD43^−^/B220^+^, and CD43^+^/B220^+^ B cell subpopulations were counted in the cells from the spleen of the Rag2/Korl and Rag2/J KO mice after staining with anti-CD43 and anti-B220 antibodies. A significant difference between the WT and Rag2 KO mice was detected in all four cell subpopulations (CD43^−^/B220^−^, CD43^+^/B220^−^, CD43^−^/B220^+^, and CD43^+^/B220^+^). Some subpopulations showed an increase in the number of cells in the Rag2 KO mice compared to WT mice, while other subpopulations showed a decrease in the number of cells in the same group. In particular, the differences between the WT and Rag2 KO mice were quite diverse between Rag2/Korl and Rag2/J KO groups. The number of the CD43^−^/B220^−^ B cell subpopulation increased 92-fold in the Rag2/Korl KO mice than in the WT mice and 14 times in Rag2/J KO mice. On the other hand, the CD43^+^/B220^−^ and CD43^+^/B220^+^ B cell subpopulations were lower (− 97.7% and − 100%, respectively) in the Rag2/Korl KO mice than the WT mice but higher (122.3% and 238.5%) in the Rag2/J KO mice (Fig. 8). Hence, there is a significant difference in the distribution of the B cell subpopulation from the spleen between the Rag2/Korl and Rag2/J KO groups.

## Discussion

Thus far, numerous mouse models with a wide range of phenotypes for human traits and diseases have been produced using various gene modification techniques [11]. The phenotypes of these models on the expression of an individual's genetic potential can be influenced by specific genetic information and environmental factors, including diet, temperature, and light cycle [12]. Therefore, some differences in the phenotypes between the model mice may exist, despite the models being produced by modifying the same gene [13]. This study compared the phenotypes of Rag2 KO mice derived from two different sources to determine the similarities and differences between their immunophenotypes. These results showed some significant differences in the immunophenotypes between the Rag2/Korl and Rag2/J KO mice, even though most of them are very similar. In addition, they will provide evidence for selecting animal models suitable for each experimental design.

The spleen is considered a critical immune organ because it helps regulate red blood cells and the immune system [14]. In this organ, old red blood cells are removed, and the protein portion of hemoglobin is metabolized to maintain the homeostasis of the body [15]. The spleen consists of two main sections: white pulp (25%) and red pulp (76–79%). Among them, the white pulp is part of the lymphatic tissue and is filled mainly with white blood cells and T cells, while the red pulp is blood-filled cavities containing some types of blood cells, including platelets, granulocytes, red blood cells, and plasma [16]. Therefore, white pulp provides lymphocytes, and red pulp filters the blood and removes foreign material and old red blood cells through the phagocytosis of macrophages [17]. Furthermore, the white pulp region of the spleen can be decreased by various physical, biological, and environmental factors. A significant decrease in white pulp was detected in the spleen of Yorkshire pigs fed with a selenium-deficient diet [18], SD rats with transient cerebral ischemia [19], and BALB/c mice that received 2-Gy ^60^Co γ-ray whole-body radiation [20]. An approximately 92% decrease in splenic white pulps was observed in the Rag2/Korl KO mice compared to the WT mice [10]. The present study examined the differences in the area of white pulp and red pulp between the WT and Rag2 KO mice to determine if there are differences between the Rag2/Korl and Rag2/J KO groups. Most of the results in the histopathological structure were similar to those of previous studies, even though the results of the present study did not calculate the exact area.

T_h_ and T_c_ cells play a key role in the cell-mediated immune response for adaptive immunity. During these processes, T_h_ cells regulate cytokine release in other immune cells, antibody class switching in B cells, and activation in T_c_ cells [21, 22]. T_c_ cells specifically kill various abnormal cells, including cancer cells, infected cells, and damaged cells, to protect the host physiological condition [23]. In Rag2 KO mice, the subpopulation of these cells changed remarkably regardless of the animal sources. Most of the CD4^+^ and CD8^+^ T cell subpopulation, including CD4^+^/CD8^−^ and CD4^+^/CD8^+^ T cells, disappeared in the spleen and thymus of the CRISPR/Cas9-mediated Rag2/Korl KO mice with the FVB and C57BL/6 background, even though there are few differences between both stains [9, 10]. A similar alteration in the subpopulation of CD4^+^/CD8^−^, CD4^−^/CD8^+^, and CD4^+^/CD8^+^ T cells was observed in the Rag2/J KO mice [7, 8]. In the present study, the difference in the CD4^+^ and CD8^+^ T cell subpopulations between WT and Rag2 KO mice was analyzed to compare the immunophenotypes between Rag2/Korl and Rag2/J KO mice. Most results of the present study were similar to several previous studies that showed significant differences in the CD4^+^ and CD8^+^ T cell subpopulation between the WT and Rag2 KO mice. On the other hand, this study presents the first evidence that there are few differences in the subpopulation of CD4^+^ and CD8^+^ T cells between the Rag2 KO mice derived from two different sources.

A remarkable difference in the number of B cell subpopulations between WT and Rag2 KO mice was observed between Rag2/Korl and Rag2/J KO groups, as shown in Fig. 8. Two subpopulations of B cells exhibited a reverse pattern in their change ratio between the Rag2/Korl and Rag2/J KO group, while the change ratio of one subpopulation was greater in the Rag2/Korl KO group than the Rag2/J KO group. These results are probably associated with the dysregulation of the maturation of B cells in the bone marrow. During maturation from lymphoid progenitor cells to mature B cells, CD43 and B220 proteins exhibit a unique expression pattern. Lymphoid progenitor cells do not express CD43 and B220 proteins (CD43^−^/B220^−^), but Pre-B and Por-B cells express these two proteins simultaneously (CD43^+^/B220^+^). As maturation progresses, the CD43 proteins disappear, and only B220 proteins remain in immature and mature B cells (CD43^−^/B220^+^) [24, 25]. Therefore, a dramatic increase in the CD43^−^/B220^−^ cell subpopulation in Rag2/Korl KO mice may associate with the complete inhibition of differentiation from lymphoid progenitor cells (CD43^−^/B220^−^) to pro-B cells (CD43^+^/B220^+^) or pre-B cells (CD43^+^/B220^+^) because the expression of these proteins was first detected in the stage of the pro-B and pre-B cells during B cell maturation [26, 27]. On the other hand, in Rag2/J KO mice, a large number (62.6%) of CD43^−^/B220^−^ cells matured into CD43^+^/B220^−^ cells even though they had not fully progressed into Pre-B and Pro-B cells. Among these cells, a small population (3.7%) of lymphoid progenitor cells progressed into pro-B cells or pre-B cells that expressed CD43 proteins. Therefore, regarding the immunology of Rag2 KO mice, they exhibited a difference in the maturation process from lymphoid progenitor cells to Pre-B and Pro-B cells. Nevertheless, to address the peculiar CD43^+^/B220^−^ cells in the spleen of Rag2/J KO derived from abnormally developed B cells, it will be important to examine what occurs to non-B and non-T populations in the spleen of Rag2 KO mice. These phenotypes in Rag2/Korl KO mice can be used in various studies for B cell-mediated immune responses. These differences in the maturation of B cells between Rag2/Korl and Rag2/J KO mice may be due to changes in the level of Rag2 gene expression based on the differences in the technique of gene deficiency and the deleting exon region when producing the mice model. Therefore, more studies will be needed to examine Rag2 expression on the surface of B cells and understand the mechanisms of action. Moreover, the lack of identification in relation to a clear cause of immunophenotypic differences should be considered a limitation of this study.

In several studies, Rag2/J KO mice showed a similar response in the immunological function and number of B cells. The decrease rates in the IgM^+^/B220^+^ cell population and the serum concentration of several Ig isotypes were significantly greater in Rag2/Korl KO mice than in the Rag2/J KO mice [9, 10]. In addition, in the Rag2/J KO mice, the splenic naive follicular B cells decreased at the age of 8–10 weeks, and more than 90% of the B cells disappeared within four months [8]. Similar decreases in the CD43^−^/B220^−^, CD43^+^/B220^−^, CD43^−^/B220^+^, and CD43^+^/B220^+^ B cell subpopulation after staining B220 and IgM antibodies were detected in the bone marrow of one-month-old Rag2/J KO mice [7].

## Conclusions

The present study compared the immunophenotypes of spleen and thymus in Rag2/Korl and Rag2/J KO mice, which were manufactured using different technologies, to determine if there are differences in the immunophenotypes for the Rag2 KO mice derived from the different sources. The differences in most immunophenotypes, including the organ weight, histopathological structure, and T cell subpopulation between WT and Rag2 KO mice were similar in the Rag2/Korl and Rag2/J KO mice, except for the differences in the subpopulation of B cells. These results will provide important information for selecting a Rag2 deficiency model in immunological research. On the other hand, further studies will be needed to expand the understanding of the causes of immunological differences and analyze other immunological factors.

## Methods

### Experimental design for animal study

The protocol to compare the immunophenotypes of Rag2 KO mice was reviewed and approved by the Pusan National University-Institutional Animal Care and Use Committee (PNU-IACUC) based on the ethical procedures for scientific care (Approval Number PNU-2021-0051). All adult mice were maintained at the Pusan National University-Laboratory Animal Resources Center, accredited by the Korea Food and Drug Administration (KFDA) (Accredited Unit Number-000231) and the Association for Assessment and Accreditation of Laboratory Animal Care (AAALAC) International (Accredited Unit Number; 001525). They were provided a standard irradiated chow diet (Samtako BioKorea Co., Osan, Korea) ad libitum, which consisted of moisture (12.5%), crude protein (25.43%), crude fat (6.06%), crude fiber (3.9%), crude ash (5.31%), calcium (1.14%), and phosphorus (0.99%). These animals were maintained in a specific pathogen-free (SPF) state under a strict light cycle (lights on at 08:00 h and off at 20:00 h) at 22 ± 2℃ and relative humidity of 50 ± 10%.

Eight-week-old Rag2/Korl KO mice with CRISPR/Cas9-mediated C3 mutant genes and WT mice (C57BL/6Korl background strain) were kindly provided by the Department of Laboratory Animal Resources (Laboratory Animals Resources Bank) at the National Institute of Food and Drug Safety Evaluation (NIFDS, Chungju, Korea). The Rag2/J KO mice and WT mice (C57BL/6J background strain) were purchased from Orient Bio Inc. (Seongnam, Korea), a local branch of Jackson Laboratory (Bar Harbor, ME, USA). The deletion of the Rag2 genes in both KO mice was determined by DNA-PCR analysis of the genomic DNA isolated from the tails of founder mice as described elsewhere [8, 9]. The Rag2 mutant genes in Rag2/Korl KO mice were amplified using two primer sets (5’-GCTGCTGCCACAATAAAGTAGTG-3’ and 5’-CCACTGTTACCATCACATTT-3’), while the targeted deletion of Rag2 gene in Rag2/J KO mice was verified using two primer sets (5’-CAGCGCTCCTCCTGATACTC-3’ and 5’-CCGCCATATGCATCCAAC-3’).

When the mice reached 16 weeks of age, they were assigned to two experimental groups: 1) Rag2/Korl KO group was composed of Rag2/Korl KO mice and WT mice (C57BL/6Korl background) and 2) Rag2/J KO group including Rag2/J KO mice and WT mice (C57BL/6J background). All mice were anesthetized by an intraperitoneal injection of Alfaxan (JUROX Pty Limited, Rutherford, Australia, 13 mg/kg body weight i.p.), after which whole blood and six organs were collected and used for further analysis.

### Quantitative reverse transcription polymerase chain reaction (RT‑qPCR) analysis

Frozen bone marrow samples were homogenized using a Polytron PT-MR 3100 D Homogenizer (Kinematica AG, Lusern, Switzerland) in RNA Bee solution (Tet-Test Inc., Friendswood, TX, USA), based on the manufacturer’s instructions. After ethanol precipitation, the total RNAs were harvested by centrifugation at 15,000 rpm for 15 min, after which the concentration was determined by Nano-300 Micro-Spectrophotometer (Allsheng Instruments Co. Ltd., Hangzhou, China). The total complementary DNA (cDNA) against mRNA was synthesized using 200 units of Superscript II reverse transcriptase (Thermo Scientific, Wilmington, DE, USA). RT-qPCR was conducted using the cDNA template obtained (2 μL), along with 2 × Power SYBR Green (10 μL; Toyobo Life Science, Osaka, Japan), and specific primers; Rag2, Forward 5’-TCTGGCCTTCAGTGCCAAAA-3’, Reverse, 5’-CACTGTTAC CATCTGCAGGG-3’. qRT-PCR was progressed for 40 cycles using the following steps: denaturation at 95°C for 15 s, followed by annealing and extension at 70°C for 60 s. The fluorescence intensity of each sample was measured at the end of the extension phase of each cycle. The cycle quantification value (Cq) was defined as described in Livak and Schmittgen’s method [28].

### Measurement of body and organs weight

The body weight of each mouse in the subset group was measured using an electronic balance (Mettler Toledo, Greifensee, Switzerland), according to the KFDA guidelines. In addition, the weights of six organs (liver, kidney, spleen, lung, thymus, and heart) collected from sacrificed WT and Rag2 KO mice were determined using the same method to measure the body weight.

### Whole blood analysis

After fasting for 8 h, all mice were anesthetized by an intraperitoneal injection of Alfaxan (JUROX  Pty Limited). The whole blood was collected from the abdominal veins using a 1 mL syringe attached to a needle (26 SWG). Each sample was placed in plain capped bottles containing ethylenediaminetetraacetate (EDTA). The components were analyzed using an automated cell counter (Beckman-Coulter Inc., Miami, FL, USA) with standard calibration, according to the manufacturer’s instructions. The levels of white blood cells (WBC), lymphocytes (LYM), neutrophil (NEU), red blood cells (RBC), hemoglobin (HGB), hematocrit (HCT), mean corpuscular volume (MCV), mean corpuscular hemoglobin (MCH), mean corpuscular hemoglobin concentration (MCHC), red cell distribution width (RDW), platelets (PLT), and mean platelet volume (MPV) were measured in duplicate for each sample.

### Histopathological analyses

The spleen and thymus were harvested from the mice of each subset group and fixed in 10% formalin solution for 48 h. The fixed tissues were embedded into paraffin blocks after trimming and sectioned into 4 µm thick slices. These sections were then stained with a hematoxylin and eosin (H&E) solution (Sigma–Aldrich, Merck KGaA, Darmstadt, Germany), and the changes in the histopathological features were examined microscopically at 40× or 400× magnification.

### Fluorescence-activated cell sorting (FACS) analysis

Briefly, the spleen and thymus were removed from anesthetized sixteen-week-old WT and KO mice to harvest lymphocytes for FACS analysis. These tissues were cut into small pieces with scissors and passed through a 70 μm cell strainer (BD Biosciences, Franklin Lakes, NJ, USA) to isolate a single-cell suspension. After removing the RBC from cell suspension using RBC lysis buffer (Sigma-Aldrich), the immune cells (1 × 10^7^ cells) were stained with PE/Cy7-anti-CD8 (100722; BioLegend, San Diego, CA, USA), FITC-anti-CD3e (152304; BioLegend), APC-anti-CD4 (100412; BioLegend), FITC-anti-CD43 (143203; BioLegend), APC-anti-CD45R/B220 (103211; BioLegend). Approximately 10,000 live cells were analyzed using a FACSCalibur™ Flow Cytometer (BD Biosciences). During these analyses, the stained cells were selected based on the forward and side scatter properties after gating for singlets. Each population was gated using the fluorescence intensity of the aforementioned antibodies against cell surface markers. The unstained cells were used to set appropriate negative gates by determining the background fluorescence levels. Single stained cells were used as a control to remove the spectral overlap between fluorophores.

### Statistical analysis

The statistical significance between each group was evaluated using a One-way Analysis of Variance (ANOVA, SPSS for Windows, Release 10.10, Standard Version, IL, USA), followed by a Tukey post hoc t-test for multiple comparisons. All values are expressed as the means ± SD. A *p*-value < 0.05 was considered significant.

## Data Availability

All the data that support the findings of this study are available on request from the corresponding author.

## Abbreviations

AAALAC: Association for Assessment and Accreditation of Laboratory Animal Care
ANOVA: Analysis of variance
AS: Atypical SCID
CIDG/AI: Combined immunodeficiency with granulomas and/or autoimmunity
EDTA: Ethylenediaminetetraacetic acid
FACS: Fluorescence-activated cell sorting
HCT: Hematocrit
HGB: Hemoglobin
H&E: Hematoxylin and eosin
Ig: Immunoglobulin
KFDA: Korea Food and Drug Administration
KO: Knockout
LYM: Lymphocytes
MCH: Mean corpuscular hemoglobin
MCHC: Mean corpuscular hemoglobin concentration
MCV: Mean corpuscular volume
MPV: Mean platelet volume
NEU: Neutrophil
NIFDS: National Institute of Food and Drug Safety Evaluation
OS: Omenn syndrome
PLT: Platelets
PNU-IACUC: Pusan National University-Institutional Animal Care and Use Committee
Rag2: Recombination activating gene2
Rag2/Korl KO: C57BL/6-Rag2^em1hwl^/Korl
Rag2/J KO: B6.Cg-Rag2^tm1.1Cgn^/J
RBC: Red blood cells
RDW: Red cell distribution width
RSS: Recombination signal sequences
RT‑qPCR: Quantitative reverse transcription polymerase chain reaction
SCID: Severe combined immunodeficiency
T_c_ cell: Cytotoxic T cell
T_h_ cell: T helper cell
WBC: White blood cell
